# Antidiabetic potential of polysaccharides from Algerian Saharan *Zygophyllum geslini* in streptozotocin-induced diabetic rats

**DOI:** 10.55730/1300-0152.2724

**Published:** 2025-01-13

**Authors:** Houria MEDJDOUB, Waffa BOUALI, Arezki AZZI, Nacéra BELKACEM, Nabila BENARIBA, Nawel MELIANI

**Affiliations:** 1 Laboratory Antifungal, Antibiotic, Physico-chemical, Synthesis and Biological Activity, Department of Biology, Faculty of Natural Sciences and Life, Sciences of the Earth and the Universe, University Abou Bekr Belkaid Tlemcen, Algeria; 2 Department of Biochemistry, College of Medicine, Imam Mohammad Ibn Saud Islamic University (IMSIU), Riyadh, Saudi Arabia; 3 Laboratory of Natural and Bioactive Products (LASNABIO), Department of Chemistry, Faculty of Sciences, Abou Bekr Belkaid University, Tlemcen, Algeria

**Keywords:** Polysaccharides extracts, pancreatic α-amylase, streptozotocin, Wistar rat, *Zygophyllum geslini*

## Abstract

**Background/aim:**

*Zygophyllum geslini*, an endemic Algerian species, has numerous properties, especially as an antidiabetic drug. In Algeria, this herb serves as condiment in Saharan dishes and as animal feed (Sheep, Goat and Camel). However, few scientific studies have reported on the medicinal properties of this Saharan species. The aims of the present work were to study 1) the chemical aspects of polysaccharides extracted from this plant, 2) their inhibitory effect on pancreatic α-amylase in vitro, and 3) their antihyperglycemic effect in streptozotocin-induced diabetic rats in vivo.

**Materials and methods:**

First, polysaccharides were extracted from *Z. geslini* aerial part (ZGAP) in hot water and precipitated with ethanol to obtain ethanolic polysaccharides and with acetone to obtain acetonic polysaccharides. The extracts were characterized using Fourier-transform infrared (FTIR) spectroscopy. In vitro antidiabetic evaluation was performed using pancreatic α-amylase, an enzyme related to diabetes. In addition, ethanolic polysaccharides were tested in vivo in streptozotocin-induced diabetic rats for 4 weeks. The rats received 100 mg/kg ZGAP ethanolic polysaccharides.

**Results:**

FTIR spectra showed that ZGAP polysaccharides were heterogeneous in composition, with extraction yield of 14.07 ± 2.61 and 4.48 ± 1.01 g/100 g of dry ZGAP and had a neutral pH (7.03 and 7.2) for ethanolic and acetonic polysaccharides, respectively. Furthermore, ZGAP polysaccharides showed potential as an α-amylase inhibitor, with IC_50_ = 3.53 ± 0.09 μg/mL for ethanolic and 7.31 ± 0.42 μg/mL for acetonic polysaccharides. Ethanolic polysaccharides were able to correct hyperglycemia caused by streptozotocin damage. A significant decrease in blood glucose levels and improvement in oral glucose tolerance were observed with ethanolic polysaccharides. Ethanolic polysaccharides extract enhanced the body weight evolution in diabetic rats.

**Conclusion:**

Based on these findings, we conclude that ZGAP polysaccharides have interesting in vivo and in vitro antidiabetic activities.

## 1. Introduction

The management of diabetes mellitus (DM) using existing therapeutic agents without any side effects remains a challenge for the modern medical system. In recent years, the search for alternative therapeutic agents for the treatment of DM has become a focus of scientific research, as medicinal plants have traditionally been used to control, manage, and/or treat DM (Ayele et al., 2021). Herbal medicines have attracted considerable interest as alternative antidiabetic remedies due to their therapeutic properties, low toxicity, and low cost (Kifle et al., 2021).

Plant mucilages and gums are polysaccharides that form a colloidal solution in water from which they can often precipitate. They are found in a widespread number of plants and in some microorganisms. They can exist either as a secondary membrane-thickening material or as intracellular substances (Amiri et al., 2021). They are used for medical and pharmaceutical purposes (Noreen et al., 2023). Recently, plant polysaccharides, especially mucilages and gums, have been reported to exhibit biological activities in humans and animals. The antitumor, immunological, anticomplementary, antiinflammatory, anticoagulant, antiviral, hypocholesterolemic, and hypoglycemic activities of a wide range of polysaccharides have been reported (Ji et al., 2021; Xue et al., 2023). Polysaccharides have been the subject of several excellent studies, both in vivo and in vitro. Currently, the identification, characterization, and evaluation of the potential health benefits of new polysaccharides from African medicinal plants have become a challenge (Addoun et al., 2021).

Allali et al. (2008) classified the plants most commonly used in northwest Algeria to treat DM. Several of these plants are recognized for their therapeutic qualities and are members of the genus *Zygophyllum*. The subject of this investigation is *Z. geslini* Coss. (Smati et al., 2004). The Algerian Sahara is home to *Z. geslini* (Zygophyllaceae), also known as Aggaya (Ozenda, 1977). In the area, this plant is referred to as an antidiabetic (Medjdoub et al., 2012; Medjdoub and Tabti, 2012), antiseptic, carminative, antibacterial, antifungal, anti-eczema, antispasmodic, and cytotoxic (Boudjelthia et al., 2017). In addition, this herb serves as a condiment in Saharan dishes and as animal feed (Sheep, Goat, and Camel). Additionally, the medicinal qualities of this species have not been extensively studied by scientists. A root dichloromethane extract was used by Smati et al. (2004) to identify an Erythrodiol with cytotoxic properties.

In the present study, we investigated the in vivo and in vitro antidiabetic effects of *Z. geslini* aerial part (ZGAP) polysaccharides. These biomolecules were extracted and characterized, and the glucose-lowering effects of the extract were evaluated in normal and streptozotocin-induced diabetic rats. Inhibition of the breakdown of polysaccharides into monosaccharides, as performed by pancreatic-amylase, is a way to decrease glucose production. To study this potential, we evaluated the inhibitory effect of the extracts on porcine pancreatic α-amylase activity in vitro.

## 2. Materials and methods

### 2.1. Plant material

**Figure 1:**
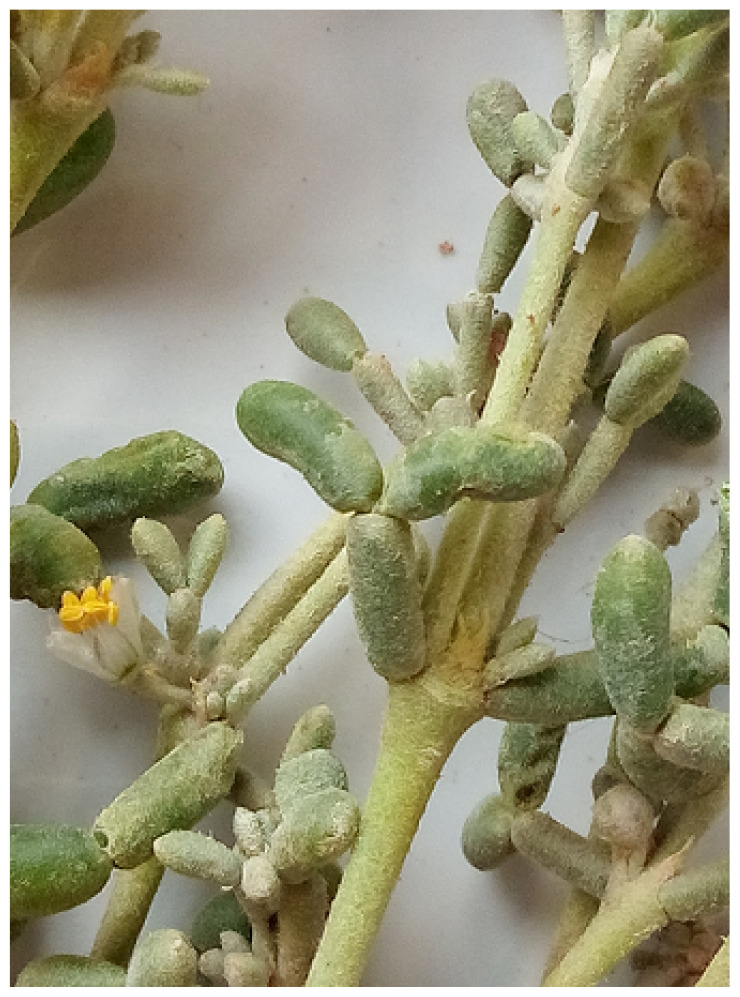
Aerial part of *Z. geslini*

Fresh ZGAP was collected from Adrar (South Algeria) in March 2020 (Figure 1). Authentication of the plant was carried out by Pr. Mahboubi A, Faculty SNV and STU (Faculty of Natural Sciences and Life, Sciences of the Earth and the Universe), University of Tlemcen, Algeria following the voucher specimen number MNHN P P00389419 deposed at the National Museum of Natural History, Paris, France. The plant material was dried at room temperature and mixed in a grinder. Powdered ZGAP was used for extraction.

### 2.2. Chemicals and reagents

Streptozotocin (STZ; S-0130, Sigma) was purchased from Sigma-Aldrich. The enzyme used is porcine pancreatic α-amylase (PPA: E.C. 3.2.1.1) in a freeze-dried form (Fluka), and its molecular weight is 13,000 Da, with a specific activity of 13 IU/mg. PPA was stored at +4 °C.

### 2.3. Extraction of polysaccharides

Polysaccharides were extracted from the plant material using the following three steps, as described in the study by Kolhe et al. (2014).

**Step 1**. Extraction of polysaccharides.

After boiling five g of ZGAP powder in 100 mL of water for 1 h, they were set aside for 2 h to complete polysaccharides extraction. The material was squeezed into a multilayer muslin cloth to remove any marks from the filtrate.

**Step 2**. Isolation of polysaccharides using ethanol.

To precipitate the polysaccharides, an equal volume of cold ethanol was added to the filtrate. The mixture was allowed for 24 h. Subsequently, the polysaccharides were precipitated via centrifugation at 3500 rpm for 20 min. The precipitate was washed with ethanol, dried in oven at approximately 45 ºC, powdered, and stored until further use.

**Step 3**. Isolation of polysaccharides using acetone.

The supernatant obtained in step 2 was used to extract acetonic polysaccharides by adding an equal volume of cold acetone to the supernatant. After 24 h, the polysaccharides were separated via centrifugation at 3500 rpm for 20 min. The precipitate was washed with acetone, dried in an oven at about 45 °C, ground into a powder, and kept in storage until needed.

### 2.4. Characterization

#### 2.4.1. Chemical analysis of ZGAP polysaccharides

The polysaccharide extracts were tested for the presence of different families of compounds (Starch, Flavonoids, Tannins, and Amino acids) according to previously described methods (Harborne, 1998). Starch iodine-test, flavonoids test by using HCl and Mg, tannins test by adding aqueous solution of FeCl_3_ and amino acids test by the reaction with alcoholic ninhydrine were done.

Proteins were quantified according to the technique employed in the study by Henry et al. (1974) based on the biuret reaction where 100 μL of aqueous polysaccharides solution were mixed with 1 mL of biuret reagent. The mixture was incubated in the dark for 30 min and absorbance was measured at 540 nm using a UV-visible spectrophometer (OPTIZENTM POP, K Lab Co., Ltd., Daejeon, Republic of Korea).

Carbohydrate content was determined as described by Dubois et al. (1956). The basic principle of this method is that carbohydrates, when dehydrated by reaction with concentrated sulfuric acid, produce furfural derivatives. The reaction between furfural derivatives and phenol results in a detectable color. In this step 1 mL of aqueous polysaccharides solution was mixed with 5 mL of concentrated sulfuric acid and 1 mL of 5% phenol aqueous solution. After incubation in dark for 30 min, absorbance was measured at 490 nm using the same UV-visible spectrophotometer.

The ash content of ZGAP polysaccharides was determined by the following (AOAC, 1990) based on mineralization by complete combustion. It is calculated as the weight of the residue (after mineralization) divided by the weight of the sample (polysaccharides extract), multiplied by 100 as a percentage.

#### 2.4.2. pH determination

Polysaccharides were weighed and dissolved in water separately to obtain a 1% w/v solution. The pH of the solution was determined using a digital pH meter (AD 1030) (Lala, 1981).

#### 2.4.3. Fourier-transform infrared (FTIR) spectroscopy

An FTIR spectrophotometer (Perkin Elmer, FT-IR Spectrum 10 software version 10.4.0) was used to obtain the infrared spectra of the polysaccharides. The samples were prepared using KBr disks. The scanning range was 400–4000 cm^−1^, and the resolution was 2 cm^−1^.

### 2.5. Pancreatic α-amylase inhibition assay

With a few modest adjustments, the Miller (1959) method was used to determine α-Amylase inhibition. 500 μL of ZGAP polysaccharides (0–5 μg/mL) or acarbose (0–100 μg/mL) in sodium phosphate buffer (0.02 M, pH 6.9) and 500 μL of sodium phosphate buffer (0.02 M, pH 6.9) containing porcine pancreatic α-amylase (E.C. 3.2.1.1) constitute the assay combination. Following that, each tube was filled with 500 μL of 1% starch solution in 0.02 M sodium phosphate buffer (pH 6.9 with 6 mmol/L NaCl).

After 15 minutes of incubation at 37 °C, a 1000 μL dinitrosalicylic acid reagent was used to stop the reaction. The mixes were then cooled after being incubated for eight min in a boiling water bath. We measured the absorbance at 540 nm. After calculation, the α-Amylase inhibitory activity was represented as a percentage (%). A control reaction was prepared as the same by replacing the inhibitor (polysaccharides or acarbose) with 500 μL of 0.02 M sodium phosphate buffer (pH 6.9).


$$\%=100\times \frac{\left(Ac-As\right)}{Ac}$$

Where Ac is the absorbance of the control reaction and As is the absorbance of the test tube with ZGAP polysaccharides or acarbose.

Tracing the linear curve of inhibitory percentage change as a function of inhibitor concentration allowed for the graphic calculation of the inhibitory concentration at 50% (IC_50_).

### 2.6. In vivo antidiabetic effect of ethanolic polysaccharides

#### 2.6.1. Animals

Three-month-old male Wistar rats weighing 230 ± 30 g were obtained from the animal house of the faculty of SNV-STU (Faculty of Natural Sciences and Life, Sciences of the Earth and the Universe), University of Tlemcen for this study. The animals were fed a laboratory animal diet and provided with water ad libitum.

#### 2.6.2. Induction of diabetes

STZ (50 mg/kg; iv) diluted in 0.1 M citrate buffer (pH 4.5) was injected into the tail vein of fasting rats to develop diabetes mellitus. Ten days following the STZ injection, fasting blood glucose levels and glycosuria were measured to confirm the presence of diabetes. The tests only included rats with positive urine glucose levels and fasting blood glucose levels of at least 200 mg/L (Crouch et al., 1978).

#### 2.6.3. Antidiabetic effect evaluation

The animals were divided into four groups of five rats each, as follows:

Normal control rats that received 0.9% saline solution (NC)

Normal rats treated with 100 mg/kg ethanolic polysaccharides (NT)

Diabetic control rats that received 0.9% saline solution (DC)

Diabetic rats treated with 100 mg/kg ethanolic polysaccharides (DT)

Using a gastric gavage needle, the animals received oral ethanolic polysaccharides for 28 days. Measurements were made of body weight, glucose levels, and the oral glucose tolerance test. The animals were given free access to water and fasted for 16 h before the experiment.

#### 2.6.4. Oral glucose tolerance test (OGTT)

After 28 days of the experiment, an OGTT was conducted. After an 18-h fast, the animals were given 3 g/kg of glucose where glucose levels were assessed at 0, 60, and 120 min (Lüllmann, 1998).

## 3. Results and discussion

### 3.1. Extraction

**Figure 2:**
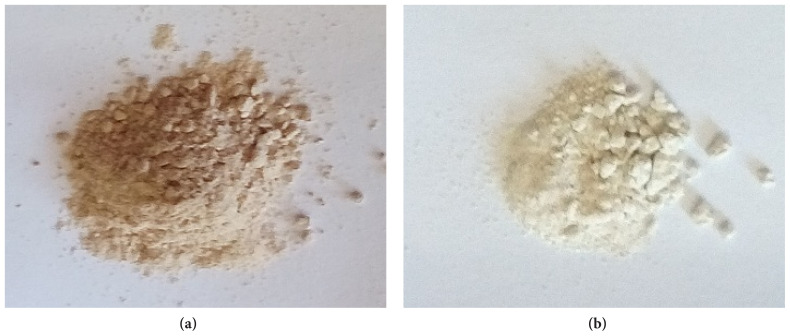
Powder aspect of ZGAP polysaccharides; (a) ethanolic polysaccharides and (b) acetonic polysaccharides

Figure 2 shows the aspect of polysaccharides obtained; both have the aspect of powder with a yellow-brown color for ZGAP ethanolic polysaccharides and white for acetonic polysaccharides. The extraction yield was 14.07 ± 2.61 and 4.48 ± 1.01 g/100 g of dry ZGAP for the ethanolic and acetonic polysaccharides, respectively (Figure 2). The ethanolic polysaccharides yield was three-fold higher than the acetonic polysaccharides yield. This is an important parameter for the in vivo ethanolic polysaccharides antidiabetic activity evaluation in Wistar rats.

The work conducted by Tan and Gan (2016) showed that *Momordica charantia*, an antidiabetic plant, is very rich in polysaccharides, with an extraction yield of 36% (Tan and Gan, 2016). In contrast, Saharan plants are salt-tolerant and grow in saline habitats. Halophytes living in extreme environments have to cope with frequent changes in salinity. This can be achieved by developing adaptive responses, including the synthesis of several bioactive molecules such as polysaccharides (Ksouri et al., 2012). In the same context, we can provide an example of *Kosteletzkya pentacarpos* (halophyte), in which the mucilage (polysaccharides) content increases in the root in response to salt stress (Zhou et al., 2021). Dhahri et al. (2023) obtained polysaccharides yield of about 5 % extracted from Ajwa, the fruit of date palm. This plant has been cultivated in dry and hot areas. Regarding these values we can conclude that polysaccharide content is variable from one plant to another.

### 3.2. Chemical analysis of ZGAP polysaccharides

The pH of 1% solution of ZGAP polysaccharides was 7.03 for the ethanolic and 7.2 for the acetonic polysaccharides (Table below). Both polysaccharides had a neutral pH, which indicates that the polysaccharides were less irritating in gastrointestinal tract (Yadav et al., 2021).

The chemical composition of both ZGAP polysaccharides showed an important difference in protein and carbohydrate content, where ethanolic polysaccharides were richer in these two compounds than acetonic polysaccharides. Regarding other constituents, both extracts were devoid of flavonoids, tannins, and starch as detailed in the table below. These results show a different composition compared with that of the mucilage of okra, which does not contain tannins or proteins (Okunlola et al., 2020).

Some polysaccharides are characterized by a heterogeneous composition and include variable contents of proteins, carbohydrates, and ash (Puligundla and Lim, 2022).

### 3.3. FTIR spectroscopy

**Figure 3:**
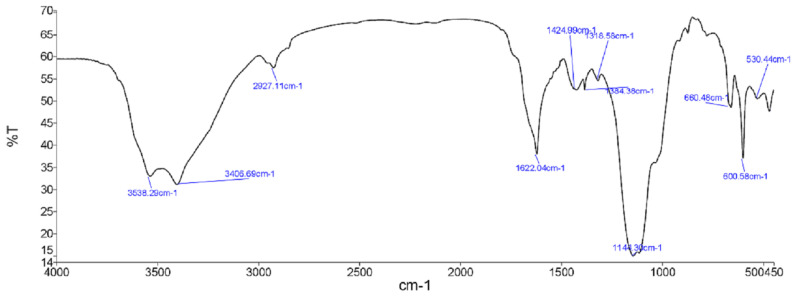
Fourier-transform infrared (FTIR) spectra of ZGAP ethanolic polysaccharides (The spectrum shows the transmittance variation with frequency of wavelength)

**Figure 4:**
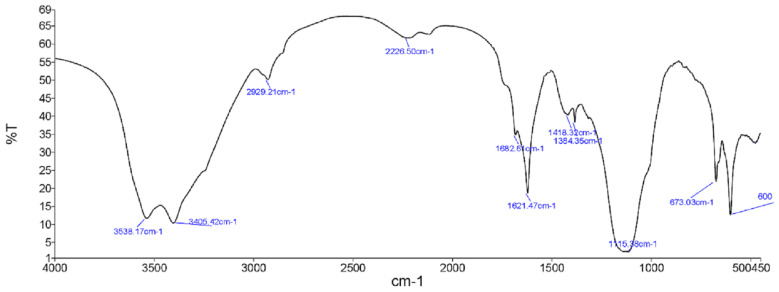
Fourier-transform infrared (FTIR) spectra of ZGAP acetonic polysaccharides (The spectrum shows the transmittance variation with frequency of wavelength)

In general, the infrared spectra of ZGAP polysaccharides as shown in Figures 3 and 4 from both extracts were similar and revealed a wide absorption band at 3400 cm^−1^, which seems to correspond to the vibration of the hydroxyl group (OH).

The wavelength of 2927 cm^−1^ seems to correspond to the stretching vibrations (C-H). The peak at 1622 cm^−1^ seems to correspond to the carboxylic anion groups (−COO^−^). This indicates the presence of both basic constituents, that is carbohydrates (region ~1150–900 cm^−1^) and proteins (region 1654–1635 cm^−1^) (Ellerbrock et al., 2019). Each particular polysaccharide has a specific band in the 1200–1000 cm^−1^ region. This region is dominated by ring vibrations overlapped with stretching vibrations of (C–OH) side groups and the (C–O–C) glycosidic band vibration (Wu et al., 2007). These results show that ZGAP polysaccharides have a heterogeneous composition which can explain the values of chemical analysis (Table).

### 3.4. Porcine pancreatic α-amylase inhibition

**Figure 5:**
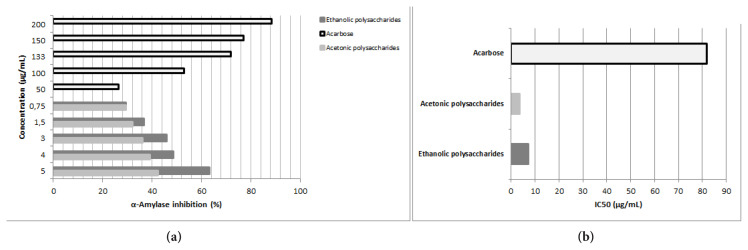
α-Amylase inhibition by ZGAP polysaccharides. (a): effect of ZGAP polysaccharides and acarbose concentrations on α-Amylase inhibition (%); (b): IC50 values are expressed in μg/mL

In vitro test was focused on PPA inhibition by the polysaccharides extracts. The results expressed in percentage and IC_50_ values are shown in Figure 5. IC_50_ is the concentration of the inhibitor (acarbose or polysaccharides) that causes 50% inhibition of α-amylase enzymatic activity. When the IC_50_ is lower, the inhibition is greater. To calculate IC_50_ numerous concentrations of each inhibitor were tested. Ethanolic polysaccharides were more effective as inhibitors than acetonic polysaccharides at the same amounts, although acarbose was less effective (Figure 5A).

The results showed a positive effect of ethanolic polysaccharides, with an IC_50_ of 3.53 ± 0.09 μg/mL. Acetonic polysaccharides had a low IC_50_ (7.31 ± 0.42 μg/mL), which indicates a good α-amylase inhibitory effect compared with that of acarbose (IC_50_ = 81.86 ± 16.86 μg/mL) (Figure 5B). The difference was considered statistically significant. The results obtained for PPA were better than the effect of the polysaccharides of *M. charantia* on the same enzyme as reported by Tan and Gan (2016). The study by Boudjelthia et al. (2017) revealed that aqueous and methanolic extracts of *Z. geslini* inhibit α-amylase with different values of IC_50_ (MeOH, IC_50_ =1.72 mg/mL; aqueous extract IC_50_=1.84 mg/ml). Furthermore, the defatted aqueous extract of the aerial part of *Z. geslini* inhibits α-amylase with an IC_50_values equal to 0.429 mg/mL (Medjdoub et al., 2023)

Our results showed that ZGAP polysaccharides had a stronger inhibitory effect on α-amylase.

### 3.5. In vivo antidiabetic effect of ethanolic polysaccharides

#### 3.5.1. Blood glucose level

**Figure 6:**
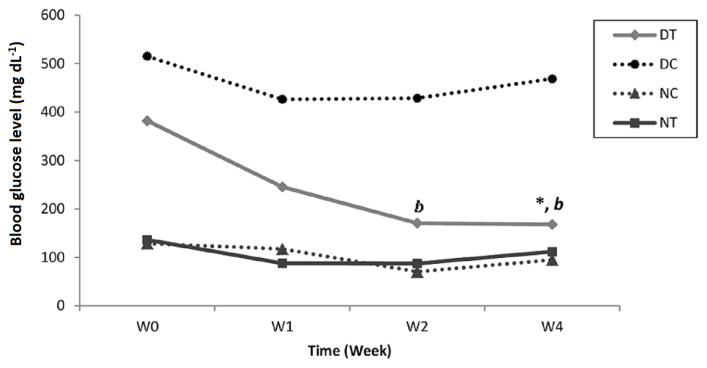
Effect of ZGAP ethanolic polysaccharides on blood glucose levels in diabetic and normal rats. bp<0.05 different from control diabetic rats. * p<0.05 different from W0 treated diabetic rats. (DT: Diabetic rats treated with 100 mg/kg ethanolic polysaccharides; DC: Diabetic control rats that received 0.9% saline solution; NT: Normal rats treated with 100 mg/kg ethanolic polysaccharides; NC: Normal control rats that received 0.9% saline solution)

Figure 6 shows the significant effect of ZGAP ethanolic polysaccharides at 100 mg/kg on blood glucose levels in diabetic rats. A positive effect was observed since the first week of treatment with the test extracts. A significant difference was observed between the control and ethanolic polysaccharides-treated diabetic groups (Figure 6). The study of Touaibia et Dhoha (2024) on *Z. geslini* aerial part revealed that animals treated with the methanol extract showed a significant decrease in blood glucose levels and glycosylated hemoglobin. In addition, a significant increase was observed in serum insulin and liver glycogen levels.

#### 3.5.2. Oral glucose tolerance test

**Figure 7:**
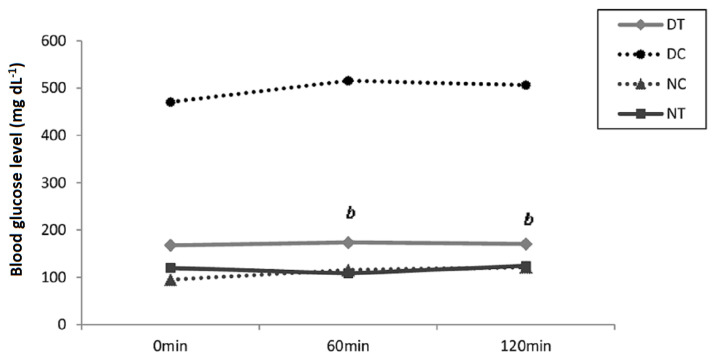
Effect of ZGAP ethanolic polysaccharides on oral glucose tolerance of diabetic and normal rats. bp<0.05 different from control diabetic rats (DT: Diabetic rats treated with 100 mg/kg ethanolic polysaccharides; DC: Diabetic control rats that received 0.9% saline solution; NT: Normal rats treated with 100 mg/kg ethanolic polysaccharides; NC: Normal control rats that received 0.9% saline solution)

OGTT was performed at the end of the experiment, after 4 weeks of treatment with the ethanolic extract of ZGAP. We tested the response of rats to a glucose load, specifically to determine whether treatment with extract at 100 mg/kg corrected the postprandial response in diabetic rats. Indeed, we observed good glucose tolerance in the treated diabetic rats. A significant difference was observed between the groups of diabetic rats (Figure 7). Medjdoub et al. (2023) had tested the effect of *Z.geslini* aerial part deffated aqueous extract on OGTT in diabetic treated wistar rats. At the same dose of 100 mg/kg this extract did not exhibit a positive effect. Regarding this research our polysaccharides show an important efficacy against diabestes induced in wistar rat.

#### 3.5.3. Body weight evolution

**Figure 8:**
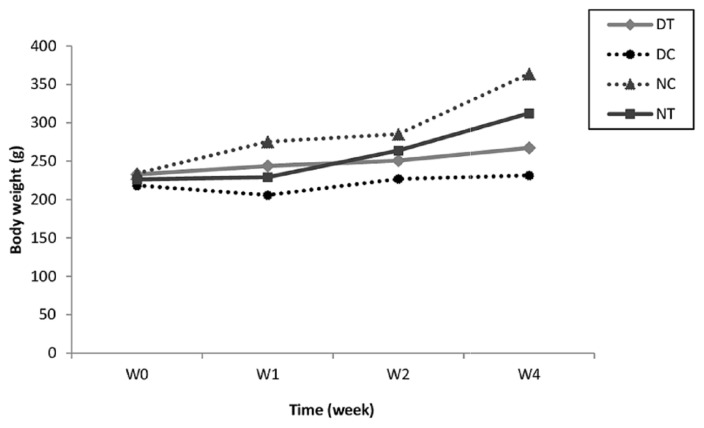
Effect extract of ZGAP ethanolic polysaccharides on body weight variation in normal and diabetic rats (DT: Diabetic rats treated with 100 mg/kg ethanolic polysaccharides; DC: Diabetic control rats that received 0.9% saline solution; NT: Normal rats treated with 100 mg/kg ethanolic polysaccharides; NC: Normal control rats that received 0.9% saline solution)

In Figure 8, we observed a normal body weight evolution in the normal groups. This confirmed that ZGAP has no toxic effects on ethanolic polysaccharides. In diabetic rats, the ZGAP ethanolic polysaccharides extract had a positive effect compared with that in untreated rats. No differences were statistically significant.

The results obtained in vivo and concerning the change in weight over 4 weeks in the treated rats indicated that 100 mg/kg ZGAP ethanolic polysaccharides were not toxic. Body weight is a good indicator of the side effects of therapeutic substances. The ethanolic and aqueous extracts of *Z. geslini* were found to have a positive effect on glycemia without toxic effects on body weight (Medjdoub and Tabti, 2012).

DM is a serious chronic metabolic disease characterized by chronic hyperglycemia (Roden and Shulman, 2019). Plant sources of antidiabetic agents have been very popular since ancient times, as they are relatively safer and much cheaper alternatives to synthetic drugs and are also mentioned in many folkloric medicines (Alam et al., 2022).

Fasting and postprandial glycemia are two important parameters in the treatment of DM (Silva et al., 2022). Regarding the evolution of glycemia, we recorded a significant improvement in fasting glycemia in treated diabetic rats. These findings show positive effects that could be explained by mechanisms at different levels, i.e., after a 16-h fast, the animal is in a postabsorptive state; any extract or molecule likely to lower blood sugar under these conditions must act by inhibiting the hepatic and renal production of glucose either directly or indirectly through the release of insulin (Yabut and Drucker, 2023).

The results on glycemia could be explained by mechanisms at different levels. On the one hand, as a potentiator of the effects of insulin or insulin mimicry; this is the case with antidiabetic drugs, of which we mainly know the class of biguanides represented by metformin (Younis et al., 2022; Albawardi et al., 2023). The latter inhibits gluconeogenesis and glycogenolysis (Johanns et al., 2023; Seube et al., 2023). On the other hand, the active substances contained in the extract may act as a stimulator of insulin secretion.

The aerial part of *Z. geslini* is rich in bioactive compounds that have been shown to have antidiabetic effects in some studies. The effectiveness of ethanolic, aqueous, and methanolic extracts varied, according to tests (Medjdoub et Tabti, 2012; Medjdoub et al., 2012; Boudjelthia et al., 2017; Touaibia and Dhoha, 2024).

## 4. Conclusion

Based on our findings, we conclude that ZGAP polysaccharides had interesting in vivo and in vitro antidiabetic effects, where ethanolic polysaccharides have an important inhibitory effect on α-amylase, which can explain their in vivo antidiabetic effect. Acetonic polysaccharides also have an inhibitory effect on α-amylase. The studied polysaccharides have a heterogeneous composition that deserves to be well developed to approach the real structure of these polymers. The current work is still a contribution, but more investigation is needed to identify the active molecule and better understand its mode of action using chemical, in vitro, in vivo, and in silico studies.

## Figures and Tables

**Table:** Chemical analysis of ZGAP polysaccharides

	pH	Proteins (g/100 g polysaccharides)	Carbohydrates (g/100 g polysaccharides)	Ash (g/100 g polysaccharides)	Starch	Flavonoïds	Tannins	Amino-acids
**Ethanolic polysaccharides**	7.03 ± 0.1	13.52 ± 8.56	23.13 ± 4.35	59.2 ± 1.34	−	−	−	++
**Acetonic polysaccharides**	7.2 ± 0.3	5.30 ± 1.66	10.27 ± 3.03	41.3 ± 2.53	−	−	−	+

+ : presence ; ++ : rich ; − : absence

## References

[b1-tjb-49-01-60] Addoun N Boual Z Delattre C Chouana T Gardarin C 2021 Beneficial health potential of Algerian polysaccharides extracted from *Plantago ciliata* Desf. (Septentrional Sahara) leaves and seeds Applied Sciences 11 9 4299 10.3390/app11094299

[b2-tjb-49-01-60] Alam S Sarker MMR Sultana TN Chowdhury NR Rashid MA 2022 Antidiabetic phytochemicals from medicinal plants: prospective candidates for new drug discovery and development Frontiers in Endocrinology 13 800714 10.3389/fendo.2022.800714 35282429 PMC8907382

[b3-tjb-49-01-60] Albawardi A Saraswathiamma D Sharma C Elomami A Souid AK 2023 Effect of Sirolimus/Metformin co-treatment on hyperglycemia and cellular respiration in BALB/c Mice International Journal of Molecular Sciences 24 2 1223 10.3390/ijms24021223 36674739 PMC9866855

[b4-tjb-49-01-60] Allali H Benmehdi H Dib MA Tabti B Ghalem S 2008 Phytotherapy of diabetes in West Algeria Asian Journal of Chemistry 20 4 2701 2710

[b5-tjb-49-01-60] Amiri MS Mohammad Zadeh V Taghavizadeh Yazdi ME Barani M Rahdar A 2021 Plant-based gums and mucilages applications in pharmacology and nanomedicine: a review Molecules 26 6 1770 10.3390/molecules26061770 33809917 PMC8004199

[b6-tjb-49-01-60] AOAC 1990 Official methods of analysis Arlington, Virginia, USA Association of official analytical chemists

[b7-tjb-49-01-60] Ayele AG Kumar P Engidawork E 2021 Antihyperglycemic and hypoglycemic activities of the aqueous leaf extract of *Rubus Erlangeri Engl*(Rosacea) in mice Metabolism Open 11 100118 10.1016/j.metop.2021.100118 34466798 PMC8384911

[b8-tjb-49-01-60] Boudjelthia Kouadri W Hammadi K Kouidri M Djebli N 2017 Evaluation of antidiabetic activity of two plants *Berberis vulgaris* and *Zygophyllum geslini* Journal of Physical Chemistry & Biophysics 7 1 1 7 10.4172/2161-0398.1000236

[b9-tjb-49-01-60] Crouch R Kimsey G Priest DG Sarda A Buse MG 1978 Effect of streptozotocin on erythrocyte and Retinal superoxide dismutase Diabetologia 15 1 53 57 150354 10.1007/BF01219329

[b10-tjb-49-01-60] Dhahri M Sioud S Alsuhaymi S Almulhim F Haneef A 2023 Extraction, Characterization, and Antioxidant Activity of Polysaccharides from Ajwa Seed and Flesh Separations 10 2 103 10.3390/separations10020103

[b11-tjb-49-01-60] Dubois M Gille KA Hamilton JD 1956 Colorimetric methods for determination of sugars and related substances Analytical Chemistry 28 3 350 356 10.1021/ac60111a017

[b12-tjb-49-01-60] Ellerbrock RH Ahmed MA Gerke HH 2019 Spectroscopic characterization of mucilage (Chia Seed) and polygalacturonic acid Journal of Plant Nutrition and Soil Science 182 6 888 895 10.1002/jpln.201800554

[b13-tjb-49-01-60] Harborne JB 1998 Phytochemical methods: a guide to modern techniques of plant analysis London Chapman and Hall

[b14-tjb-49-01-60] Henry R Cannon DC Winkelman JW 1974 Clinical chemistry: principles and technics Hagerstown, MD Harper and Row, London

[b15-tjb-49-01-60] Ji X Peng B Ding H Cui B Nie H 2021 Purification, Structure and Biological Activity of Pumpkin Polysaccharides: A Review Food Reviews International 39 1 307 319 10.1080/87559129.2021.1904973

[b16-tjb-49-01-60] Johanns M Hue L Rider MH 2023 AMPK inhibits liver gluconeogenesis: fact or fiction? Biochemical Journal 480 1 105 125 10.1042/BCJ20220582 36637190

[b17-tjb-49-01-60] Kifle ZD Bayleyegn B Tadesse TY Woldeyohanins AE 2021 Prevalence and associated factors of herbal medicine use among adult diabetes mellitus patients at government hospital, Ethiopia: an institutional-based cross-sectional study Metabolism Open 11 100120 10.1016/j.metop.2021.100120 34485891 PMC8403751

[b18-tjb-49-01-60] Kolhe S Kasar T Dhole SN Upadhye M 2014 Extraction of mucilage and its comparative evaluation as a binder American Journal of Advanced Drug Delivery 2 3 333 343

[b19-tjb-49-01-60] Ksouri R Ksouri WM Jallali I Debez A Magné C 2012 Medicinal halophytes: potent source of health promoting biomolecules with medical, nutraceutical and food applications Critical Reviews in Biotechnology 32 4 289 326 10.3109/07388551.2011.630647 22129270

[b20-tjb-49-01-60] Lala PK 1981 Practical pharmacognosy Calcutta Lina Guha

[b21-tjb-49-01-60] Medjdoub H Tabti B 2012 Antidiabetic effect of the aerial part ethanolic extracts of *Zygophyllum geslini* Coss. in streptozotocin induced-diabetic rats Metabolic and Functional Research on Diabetes 5 17 20

[b22-tjb-49-01-60] Medjdoub H Tabti B Baatouche M Baou L Zehhaf S 2012 Antihyperglycemic effect of *Zygophyllum geslini* aqueous extract in streptozotocin-induced diabetic wistar rats Journal of Life Sciences 6 6 652

[b23-tjb-49-01-60] Medjdoub H Bouhada Y Boufeldja T 2023 Antidiabetic Effect of *Zygophyllum geslini* aerial Part: in vivo and in vitro studies Phytothérapie 21 4 194 198 10.3166/phyto-2022-0372

[b24-tjb-49-01-60] Miller GL 1959 Use of dinitrosalicylic acid reagent for determination of reducing sugar Analytical Chemistry 31 3 426 428

[b25-tjb-49-01-60] Noreen S Hasan S Ghumman SA Anwar S Gondal HY 2023 Formulation, statistical optimization, and in vivo pharmacodynamics of *Cydonia oblonga* mucilage/alginate mucoadhesive microspheres for the delivery of metformin HCl ACS Omega 8 6 5925 5938 10.1021/acsomega.2c07789 36816641 PMC9933240

[b26-tjb-49-01-60] Okunlola A Odeniyi MA Arhewoh MI 2020 Microsphere formulations of ambroxol hydrochloride: influence of Okra (*Abelmoschus esculentus*) mucilage as a sustained release polymer Progress in Biomaterials 9 1 65 80 10.1007/s40204-020-00132-5 32504415 PMC7289913

[b27-tjb-49-01-60] Ozenda P 1977 Flore du Sahara Paris Edition du centre national de la recherche scientifique

[b28-tjb-49-01-60] Puligundla P Lim S 2022 A review of extraction techniques and food applications of flaxseed mucilage Foods 11 12 1677 10.3390/foods11121677 35741874 PMC9223220

[b29-tjb-49-01-60] Roden M Shulman GI 2019 The integrative biology of type 2 diabetes Nature 576 7785 51 60 10.1038/s41586-019-1797-8 31802013

[b30-tjb-49-01-60] Seube PA Djerada Z Konecki C Dupont V Goury A 2023 Simultaneous, dual continuous veno-venous haemofiltration for refractory metformin-induced lactic acidosis: a case report Clinical Toxicology 61 2 134 136 10.1080/15563650.2022.2163900 36735329

[b31-tjb-49-01-60] Silva ML Bernardo MA Singh J de Mesquita MF 2022 Cinnamon as a complementary therapeutic approach for dysglycemia and dyslipidemia control in type 2 diabetes mellitus and its molecular mechanism of action: A review Nutrients 14 13 2773 10.3390/nu14132773 35807953 PMC9269353

[b32-tjb-49-01-60] Smati D Longeon A Guyot M 2004 3 β-(3, 4-Dihydroxycinnamoyl)-erythrodiol, a cytotoxic constituent of *Zygophyllum geslini* collected in the Algerian Sahara Journal of Ethnopharmacology 95 2–3 405 407 15507367 10.1016/j.jep.2004.08.011

[b33-tjb-49-01-60] Tan HF Gan CY 2016 Polysaccharide with antioxidant, α-amylase inhibitory and ACE inhibitory activities from *Momordica charantia* International Journal of Biological Macromolecules 85 487 496 10.1016/j.ijbiomac.2016.01.023 26778156

[b34-tjb-49-01-60] Touaibia M Dhoha A 2024 Biochemical characterization, antioxidant and antidiabetic properties of *Zygophyllum geslini* Coss extracts International Journal of Biological and Chemical Sciences, 18 3 1105 1114 10.4314/ijbcs.v18i3.30

[b35-tjb-49-01-60] Wu Y Cui SW Tang J Wang Q Gu X 2007 Preparation, partial characterization and bioactivity of water-soluble polysaccharides from boat-fruited sterculia seeds Carbohydrate Polymers 70 4 437 443 10.1016/j.carbpol.2007.05.010

[b36-tjb-49-01-60] Xue T Ruan K Tang Z Duan J Xu H 2023 Isolation, structural properties, and bioactivities of polysaccharides from *Althaea officinalis* Linn.: A review International Journal of Biological Macromolecules 242 125098 10.1016/j.ijbiomac.2023.125098 37245776

[b37-tjb-49-01-60] Yabut JM Drucker DJ 2023 Glucagon-like peptide-1 receptor-based therapeutics for metabolic liver disease Endocrine Reviews 44 1 14 32 10.1210/endrev/bnac018 35907261

[b38-tjb-49-01-60] Yadav A Chaudhari P Khatri S Thapa C 2021 Formulation, in-vitro evaluation and comparative study of itopride hydrochloride loaded sustained release matrix tablet using Okra mucilage as a natural binder Tablet 13 25 10.47583/ijpsrr.2021.v68i02.008

[b39-tjb-49-01-60] Younis HY Thanoon IA Fadhil NN Merkhan MM 2022 Effect of zinc as an add-on to metformin therapy on glycemic control, serum insulin, and C-Peptide levels and insulin resistance in type 2 diabetes mellitus patient Research Journal of Pharmacy and Technology 15 3 1184 1188 10.52711/0974-360X.2022.00198

[b40-tjb-49-01-60] Zhou M Lutts S Han R 2021 *Kosteletzkya pentacarpos*: a potential halophyte candidate for phytoremediation in the Meta (loid) s polluted saline soils Plants 10 11 2495 10.3390/plants10112495 34834857 PMC8624882

